# Metaphor in psychosis: on the possible convergence of Lacanian theory and neuro-scientific research

**DOI:** 10.3389/fpsyg.2015.00664

**Published:** 2015-06-03

**Authors:** Michele Ribolsi, Jasper Feyaerts, Stijn Vanheule

**Affiliations:** ^1^Clinica Psichiatrica, Dipartimento di Medicina dei Sistemi, Università degli Studi di Roma Tor Vergata, Rome, Italy; ^2^Child Neuropsychiatry Unit, Neuroscience Department, “Children’s Hospital Bambino Gesù”, Research Hospital, Rome, Italy; ^3^Department of Psychoanalysis and Clinical Consulting, Ghent University, Ghent, Belgium

**Keywords:** schizophrenia, psychoanalysis, right hemisphere, psychosis, symbolization, metaphor, concretism, Lacan

## Abstract

Starting from the theories of leading psychiatrists, like Kraepelin and de Clérambault, the French psychoanalyst Jacques Lacan (1901–1981) formulated an original theory of psychosis, focusing on the subject and on the structuring role of language. In particular, he postulated that language makes up the experience of subjectivity and that psychosis is marked by the absence of a crucial metaphorization process. Interestingly, in contemporary psychiatry there is growing empirical evidence that schizophrenia is characterized by abnormal interpretation of verbal and non-verbal information, with a great difficulty to put such information in the appropriate context. Neuro-scientific contributions have investigated this difficulty suggesting the possibility of interpreting schizophrenia as a semiotic disorder which makes the patients incapable of understanding the figurative meaning of the metaphoric speech, probably due to a dysfunction of certain right hemisphere areas, such as the right temporoparietal junction and the right superior/middle temporal gyrus. In this paper we first review the Lacanian theory of psychosis and neuro-scientific research in the field of symbolization and metaphoric speech. Next, we discuss possible convergences between both approaches, exploring how they might join and inspire one another. Clinical and neurophysiological research implications are discussed.

## Introduction

Seminal descriptions of schizophrenia stress that next to symptoms like hallucinations and delusions, remarkable alterations can be observed in patients’ mental life. For example, concerning dementia praecox Kraepelin (1913, p. 3) accentuated a “peculiar destruction of the internal connections of the psychic personality,” as a result of which emotional and volitional aspects of mental life get deregulated. Bleuler (1934), for his part, stressed that disturbances of association make up an especially important basic symptom, and indicated that these result in the loss of formal coherence of speech and in disconnected thinking. Since Kraepelin and Bleuler, many authors have tried to characterize the “core feature” of schizophrenia, on which all the symptoms depend. For example, Parnas (2011) suggested that it is marked by autism, i.e., a change in the structures of subjectivity, anomalies of self-experience, disturbed relations to the world, solipsism and isolation. Others have speculated that a pivotal role may be played by negative symptoms or by neurocognitive deficits (Remington et al., 2011).

A prominent yet underestimated symptom of schizophrenia is the peculiar interpretation and processing of verbal and non-verbal information, with a great difficulty of patients to put these in the appropriate context. Some authors have investigated this difficulty suggesting the possibility of interpreting schizophrenia as a semiotic disorder, which disables patients ability to understand the figurative meaning of metaphoric speech (Harrod, 1986). Next to that, psychoanalytic authors, like Jacques Lacan, have suggested that psychosis may be explained in terms of a problem at the level of subjective representation by means of language, which led him to stress a fundamental problem at the level of metaphor use.

This paper has a double focus. On the one hand we discuss Jacques Lacan’s thesis concerning the structure that underlies psychotic functioning. Lacan starts from a unified psychosis concept, in which different forms of psychosis, like schizophrenia and paranoia are considered to be related to a central problem of metaphor use at the level of subjectivity. On the other hand we discuss neuro-scientific literature concerning the right hemisphere-dependent impairment in processing metaphoric speech, which characterizes schizophrenia, and highlight the roles of symbolization and metaphor comprehension in the development of psychotic symptoms. In a final step we explore how both approaches might converge and inspire one another. We will start with a broad contextualization of Lacan’s work, bringing us to his concept of the divided subject.

## Lacan on Psychosis

French psychoanalyst Jacques Lacan’s oeuvre covers a period of over 50 years, during which time he developed a theory that innovated psychoanalytic practice and introduced a new method of reflecting on human subjectivity.

Lacan’s work is often divided into three periods: the Imaginary (1936–1952), the Symbolic (1953–1962), and the Real (1963–1981), as he reported in the Seminar R.S.I. (1974–1975): “I began with the Imaginary, I then had to chew on the story of the Symbolic […] and I finished by putting out for you this famous Real.” The Symbolic is the register of symbolic language, the signifying chain of words, while the Imaginary is related to the image of each individual as an object in the mirror, and to the image one has of others. Normally, all perception is filtered through the registers of the Imaginary and the Symbolic. On the contrary, the Real is “the thing in itself,” it is the un-Imaginable and un-Symbolizable.

While most of Lacan’s work is not specifically concerned with psychosis, the question of psychosis was addressed in various papers and during several seminars held between the early 1950s and the late 1970s. During the nineteenth and early twentieth centuries psychosis was largely understood in terms of organic deficiencies: psychotic symptoms were seen as a surface phenomena of presumed underlying disturbances in the brain. While Lacan was interested in the strict neurobiological work of his forbearers, particularly because of their detailed observation of psychotic patients’ functioning, he believed that these theories overlooked the complexity of the psychotic experience. A main issue that remained neglected was the question as to how psychosis is intertwined with subjectivity.

Four periods can be discerned in Lacan’s work on psychosis (Vanheule, 2011). These periods are not strictly separated, but such a distinction helps us grasp the different emphasis Lacan put on different concepts across time.

The first period concerns Lacan’s work in the 1930s and 1940s, and focuses on identification. At that moment Lacan brings psychoanalysis into dialog with theories of leading psychiatrists, like Kraepelin and Jaspers, and gradually differentiates the psychoanalytic perspective from the psychiatric one. He proposes that psychosis is characterized by a specific type of identification: the ego is captured by an ideal image, from which it is not well differentiated. This results in confusion and suspicion.

In the 1950s (second period) he puts this view aside, contending that what matters in psychoanalysis is the materiality of speech. He then argues that language makes up the experience of subjectivity and that psychosis is marked by a deficiency in certain metaphor uses. Central to this period is his year-long seminar on psychosis (Seminar III; Lacan, 1955–1956), which gave rise to the text *On a Question Prior to Any Possible Treatment of Psychosis* (Lacan, 1959–2006). During this time Lacan provided his most extensive discussion of the topic of psychosis, proving himself a truly innovative thinker. His re-interpretation, or “structural analysis” (Lacan, 1959–2006, p. 449), of Schreber’s (1955) autobiography is crucial in this phase of his work.

However, as his seminar progressed Lacan revised these ideas twice. From his 10th seminar onward (1962–1963; third period), Lacan stressed that psychosis also entails a different experience of corporeality and a different relation to the libidinous drive. He then embraces the thesis that some aspects of being cannot be grasped via language. The two key-concepts used to address this domain of being are “jouissance” and the “object *a.*” The fourth era in Lacan’s work on psychosis gravitates around his 23rd seminar (Lacan, 1975–1976) where knot theory is used to operationalize mental functioning. The central question he then works with concerns how a *link* can be made in the relation between the three registers that make up mental life: the Real, the Symbolic and the Imaginary.

While Lacan’s work from the 1960s and 1970s adds important aspects to his psychosis theory, his ideas of the 1950s are usually seen as truly groundbreaking (Nobus, 2000; Fink, 2007). Inspired by the structural linguistic works of de Saussure (1916), Benveniste (1966), and Jakobson (1971–1985) he then respectively pays attention to the role and function of signifiers, pronouns and metaphors in psychosis. At the same time Lacan starts rebelling against mainstream psychodynamic interpretations of psychosis, which focus too strongly on intrapsychic disturbances.

## Language and the Subject

Guided by a return to the work of Freud, in the 1950s Lacan proposed that Freud’s ideas on the functioning of the unconscious, and more broadly of mental life, can be better understood within the context of linguistic theory: desire is structured by language and expressed in speech (Lacan, 1953). From this perspective, the unconscious is made up of so-called signifiers, which, following de Saussure (1916) and Lacan (1957–2006) considers as the most elementary units of language. Indeed, just like de Saussure, Lacan makes a distinction between signifier and signified. The signified concerns the concept or the idea that the linguistic sign conveys, while the signifier refers to the “acoustic image” or the “sound image” that makes up the sign (de Saussure, 1916, p. 66). In the processes of speech and writing, signifiers are related to signifieds, which results in the creation of signification: “A signifying unit presupposes the completion of a certain circle that situates its different elements [signifier and signified]” (Lacan, 1955–1956, p. 263).

de Saussure (1916) proposes that in human language use of the signified predominates over the signifier. This accent on the concept or the idea assumes that language is used to transfer and express meaning. Lacan (1957–2006), by contrast, clearly argues for the opposite, and considers the signifier as primordial to the signified. He argues that at the level of the unconscious, the signifier predominates and even makes up the logic of how symptoms are organized.

Another idea Lacan adopts from de Saussure (1916) is that the moment language is used in speech signifiers are linked in signifier-to-signifier connections or chains. Signifiers connect in “signifying chains” says Lacan (1957–2006, p. 418), like links in a necklace. Both Roman Jakobson and Lacan follow de Saussure in this idea. Jakobson (1953–1971, p. 565) concludes that the principle of connecting signifiers makes up the syntagmatic axis in language: “syntax is concerned with the axis of concatenation.” In a broader time frame these signifying chains make up the *diachrony* of language. This means that by linking signifiers in linear chains the dimension of *time* is introduced. On the one hand adding a new signifier implies that preceding signifiers obtain a status of anteriority. On the other hand the linear connection of signifiers in speech is open-ended and leads to the anticipation of additional signifiers. The articulation of each signifier sets up the expectation of yet another signifier that will further complement the chain.

Next to the syntagmatic or diachronic dimension in language, de Saussure and Jakobson also distinguish a so-called associative or synchronic axis. Jakobson (1953–1971) refers to this as the dimension of *synchrony*, which he opposed to diachrony. Synchrony means that when a signifier is diachronically chained in speech, it simultaneously echoes many other signifiers that are not actually articulated, but are connected to it associatively.

Furthermore, Jakobson (1953–1971)^1^ connects diachrony and synchrony to two characteristic figures of speech: metaphoric substitution takes places at the level of synchrony, and diachrony is most characteristic of metonymy. Lacan (1957–2006) especially focused on these processes of metaphor and metonymy, suggesting that they correspond with condensation and displacement, the two main processes Freud discerned at the level of the unconscious. Condensation is understood in terms of the metaphoric process, and displacement in terms of metonymy (Vanheule, 2011).

**FIGURE 1:**
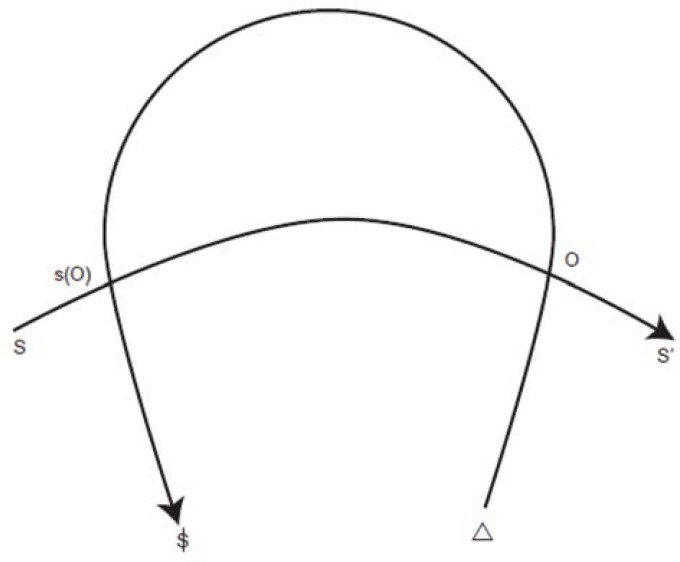
**The logic of signification**.

With the concepts of diachrony and synchrony the process of signification is also specified (Van Haute, 2001; Naveau, 2004). Signification is not merely an effect of linking signifiers to signifieds, but is created as an effect of syntactic processes in the signifying chain. Figure 1 illustrates Lacan’s understanding of how the logic of signification is generated in language (Lacan, 1957–1958, 1960–2006; see also “Graph I” in Lacan, 1960–2006, p. 681).

In Figure 1, the horizontal arrow from S to S′ indicates how, on the one hand, speech is always a matter of linking signifiers in a chain; a metonymic process that temporally follows diachronic logic. On the other hand, speech will only be generated if a person feels the urge to articulate an intention or need, symbolized by Δ at the basis of the returning arrow. The returning arrow indicates how this intention eventually leads to the production of *meaning* and *subjectivity*. The two intersections between the arrows are thereby crucial.

The right intersection (indicated as O) refers to the Other or “the locus of the treasure trove of signifiers” (Lacan, 1960–2006, p. 682), and indicates that in the production of speech, signifiers are picked up from the lexicon we have at our disposal. The idea of treasure trove thereby expresses synchrony: in the Symbolic all signifiers are simultaneously given. A speaker picks up elements from this whole and links them in a chain. By doing so the anticipation of meaning begins. Indeed, through the use of signifiers, an idea is expected to arise. Yet as long as the advent of a meaning is under construction, it is suspended (Lacan, 1959–2006, p. 446).

The left intersection refers to the moment in which the intention to speak eventually crystallizes in signification; in a message, indicated as s(O) in the figure above. Lacan stresses that, at a temporal level, punctuation follows a *retroactive* logic. It is only if a sufficient number of signifiers have been articulated that meaning can arise. Signifiers that are articulated later will thereby determine the final meaning of formerly uttered signifiers.

Finally, and most importantly, the process of generating meaning also has the effect of producing subjectivity: via language use the *subject* takes shape, indicated as $ in Figure 1. Figure 1 shows that whereas Lacan frequently made use of linguistic concepts, his primary aim is clinical and directed toward insight into how subjectivity is generated via the use of signifiers. In this respect personal pronouns occupy an important role. Because of our use of personal pronouns speech turns into a self-referential process: as we speak we outline who we are. However, the signifiers articulated in the signifying chain mark and connote the speaker, but never denote the speaker exactly. Lacan considers the subject as an effect of this connotation and thus concludes that the subject is only half-said, which is expressed by the bar through S in the symbol for the subject: $. In terms of the process of signification, $ is the result of a dialectical tension. At the level of the message, speech functions to build images regarding who we are. These images make up the ego, but are selective imaginary self-representations that exclude certain signifiers. The unconscious subject consists of these “forgotten” signifiers, across which the subject is fundamentally scattered; hence the idea that the subject is divided (Lacan, 1957–1958, 1960–2006). In neurosis this division is experienced as internal, hence the neurotic tendency to repress, whereas in psychosis it is experienced as disconnected from one’s own intentions and as coming from without.

## Metaphorization in Psychosis

Following his discussion on the divided subject and the signifier, Lacan examined the structure of psychosis. He suggests that in psychosis a specific signifier, which concerns both the law and naming, is absent and, as a result, the structure of the Symbolic is unstable. He calls this signifier the “Name-of-the-Father,” and refers to its absence with the concept of “foreclosure.” Both of these concepts were introduced during his discussion of Freud’s Oedipus complex. On the one hand Lacan considered Freud’s account of the Oedipus complex as important for characterizing the structure of psychopathology. On the other hand he believed it gave too mythical account of this transition. Whereas Freud mainly described the Oedipus complex in developmental terms, Lacan (1959–2006) believes that what actually takes place is a metaphorical transition. In his interpretation a special signifier is installed during the Oedipus complex: the paternal signifier or Name-of-the-Father. This signifier nominates the desire of the maternal figure with which the child is first confronted, and it opens up the dimension of the law. Through the Name-of-the-Father people understand themselves and others in terms of rules and standards that one should obey. They use this signifier to make sense of desire and it helps them to experience permanency in social relations.

Roman Jakobson was a crucial source of inspiration for Lacan’s theory of the foreclosure of the Name-of-the-Father. It is via Jakobson, Lacan became interested in the structure of metaphor. Actually, Jakobson extensively discussed two linguistic tropes: metonymy and metaphor.

*Metonymy* takes place within the diachronic linking of signifiers in a chain, where one signifier evokes another because of a thematic connection at the level of the signified. For example, in the phrase: “I sent her an e-mail.” The words “sent” and “e-mail” are thematically related and are connected in a relation of continuation. A characteristic of metonymy is that the signifieds communicated are in line with each other and provide a coherent context. Such thematic connection entails a mental experience of continuity. Indeed, a sense of continuity is experienced because we produce ideas that are thematically linked. Through metonymy the speaking subject is connoted: As we integrate personal pronouns like “I” and “her” in our speech, metonymy presents the subject, without actually defining who the subject is.

*Metaphor* is different. Jakobson (1953–1971) explained metaphor as a process at the level of synchrony, in which one signifier is replaced by another based on similarity. Overall, Lacan follows Jakobson’s idea, and formalizes it as follows (Lacan, 1959–2006, p. 464):

**Figure:**
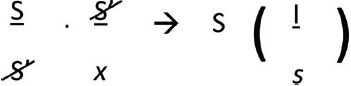


The formula indicates that metaphor is a process in the signifying chain, indicated by the dot between the two fractions in the left section of the formula, in which a signifier S replaces another signifier that was not uttered but was metonymically anticipated in the signifying chain, expressed by the barred S′. The *x* from the formula symbolizes metonymic anticipation of signification. The signifier S that is actually produced in a metaphor is surprising in a given speech context, and disrupts the metonymic process: at the level of the signified metaphors produce discontinuity. Metaphors create shifts in meaning, and add new ideas to a line of reasoning. Indeed, the effect of a metaphoric substitution between signifiers is an induction of meaning (see Lacan, 1959–2006, p. 465). In this formula the symbol “I” represents the “impact of the signifier on the signified” (Lacan, 1957–1958, p. 428), and “*s*” refers to the signified on which an influence is exercised^2^.

Just like metonymy, for Lacan metaphor is not simply a linguistic trope, but a mode of speech along which the subject is defined. The effect of metaphor is such that the subject, which was connoted until then, is denoted and identified. At the level of meaning, a metaphor attributes predicates or characteristics to the subject, and tells us something about the identity of the person that is presented via speech.

As indicated, Lacan suggests that the structure underlying the Oedipus complex concerns a process of metaphorization, of which *naming* is a crucial component. Oedipal dynamics can be adequately characterized by what he calls “the metaphor of the Name-of-the-Father” (Lacan, 1959–2006, p. 465) or the “paternal metaphor” (Lacan, 1959–2006, p. 463). To highlight the structure of the metaphor of the Name-of-the-Father Lacan (1959–2006, p. 465) uses the following formula:

**Figure:**
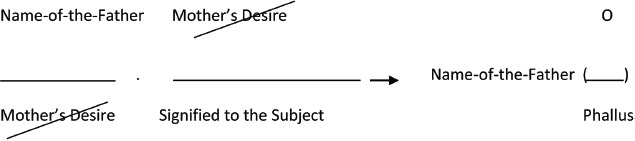


The formula indicates that initially the child has a representation of what the primordial other (mother) does—she appears and disappears over and again—but cannot make sense of what she actually wants. The child has a signifier for the mother, but no signified for her desire: it is unclear what she wants. The child first deals with this uncertainty by believing that (s)he is the central point around which the mother’s desire is turning. In Lacan’s (1956–1957, 1958) terminology the child is the so-called “imaginary phallus” for the mother, or the signified of her desire. This belief entails both primary narcissism and rivalry, which are transcended by the installment of the Name-of-the-Father. In the paternal metaphor, the signifier of the Name-of-the-Father substitutes that of the Mother’s Desire, and leads to the creation of new signification. The Name-of-the-Father *names* maternal desire, expressed by the erasure in the formula. The paternal signifier comes as a substitute for the maternal signifier and along this way desire is subjected to the law, i.e., to social and cultural conventions. The Name-of-the-Father is the signifier of culture and taboo. By replacing the signifier of maternal desire with the Name-of-the-Father, maternal desire loses its enigmatic quality: meanings get attributed to the primordial other’s actions.

A further effect of this naming is that a space for symbolic identification is created, i.e., a type of identification that is guided by signifiers and that concerns his/her position toward desire. By installing the paternal signifier, maternal desire gets framed in terms of patterns and laws of transaction. Apart from her maternal position the mother also occupies other roles, for example the position of wife etc., and in addition to her interactions with the child she is engaged in many other exchange relationships that the child has nothing to do with. At this stage the observation that the mother’s desire is not directed solely to the child gives rise to the question of what organizes maternal desire. The answer to this question is “the Phallus” or the “symbolic Phallus.” In Lacan’s (1959–2006) theory the concept of the symbolic Phallus is a synonym for the ultimate characteristic that makes the object of maternal desire desirable. With the aim of positioning itself within the (m)other’s desire, the child will detect these characteristics and identify with them.

Additionally, Lacan suggests that the Name-of-the-Father changes the status of the Other for the subject. In his article *On a Question Prior to any Possible Treatment of Psychosis*, Lacan gives quite a specific interpretation to the concept Other, defining it as “the locus from which the question of his [the subject’s] existence may arise” (Lacan, 1959–2006, p. 459). The idea is that at the level of the unconscious each speaking subject is confronted with a basic question concerning its own identity as subject: “Who am I?” More precisely this question relates to three issues: one’s “sex,” one’s “contingency in being” (Lacan, 1959–2006, p. 459), and “the relational signifiers of love and procreation” (Lacan, 1959–2006, p. 461). The question of *the subject’s sex* concerns whether one is a man or a woman, as well as the issue of how one gives shape to sexual identity. The matter of *contingency in being* refers to the fortuity of life, and to the question what life means in the light of death. The *relational signifiers* in their turn point to the question of what it is that really connects people in love, and to the question of parenthood. Indeed, in the 1950s Lacan principally discusses the question of subjectivity in terms of the effect of questions on the subject. Such questions are thought to be fundamental to human existence and expressed clinically in symptoms, rather than in self-conscious questions.

Lacan’s formula of the paternal metaphor indicates that at the level of the unconscious answers to questions of the existence of the subject cannot be found. However, the incorporation of the Name-of-the-Father provides a framework to address these. Indeed, the Name-of-the-Father provides a symbolic structure to construct answers to these questions. In principal this is done via symbolic identification. People answer questions of their own existence by adopting characteristics they assume make them desirable to others (phallic traits). This is what the last part of Lacan’s formula expresses: because of the Name-of-the-Father, the Phallus is the common denominator to all questions on the subject at the level of the Other.

In neurosis the signifier of the Name-of-the-Father replaces the signifier of maternal desire, such that a dialectical identity is inaugurated. Incompatible identifications are disagreeable to the unity-seeking ego and are repressed (Nobus, 2000; Verhaeghe, 2004; Ver Eecke, 2006). In psychosis the situation is different since the signifier of the Name-of-the-Father remains absent. Lacan (1959–2006) states: “At the point at which the Name-of-the-Father is summoned… a pure and simple hole may thus answer in the Other” (pp. 465–466). To refer to the absence of the Name-of-the-Father Lacan uses the concept of *foreclosure*. The result of such foreclosure is that the paternal metaphor is not set in motion.

At the level of subjectivity, such non-installment of the Name-of-the-Father has drastic effects: the articulation of the subject is rendered chaotic; the subject is not named in relation to maternal desire; and in relation to questions of existence a gaping hole remains. Due to foreclosure the questions of existence at the level of the unconscious—“Who am I?” and “What do you want from me?”—cannot be addressed in a conventional manner. The questions that typically lead to the articulation of the subject do not obtain an answer, which destabilizes the experience of identity. This implies that the desire of the (m)other remains fundamentally enigmatic. In psychosis the “code of convention” that is needed to navigate the enigma of what the other wants is absent. The effect is a fundamental difficulty in making sense of the other’s intentions, as well as in experience a feeling of identity in relation to questions of the existence of the subject. Indeed, with respect to personal identity foreclosure implies that a framework for addressing questions of existence remains lacking. The result is that there is little to hold on to vis-à-vis one’s identity as a man or a woman, how to deal with love and sexuality, how to give shape to intergenerational relationships, or the purpose of life in the light of death. As a result of foreclosure, questions on the existence of the subject reveal what Lacan calls “the Real,” that is, the realm of the radically enigmatic non-signified.

## Language Use in Psychosis: Implications of Lacan’s Foreclosure Theory

While highly conceptual in nature, Lacan’s theory of psychosis has clear clinical implications, entailing several hypotheses on the functioning of psychotic patients. Above all, the unconscious has a different status than in neurosis. Foreclosure, writes Lacan (1959, p. 465) refers to “a function of the unconscious that is distinct from the repressed.” Situations in which a person is confronted with questions on the existence of the subject are Real. In psychosis these questions don’t give rise to metaphoric generation of meaning, nor to a feeling of identity. They puzzle and overwhelm the psychotic person, and eventually give rise to decompensation. We believe that with respect to psychotic language use, Lacanian theory entails, at least, three precise ideas.

A first phenomenon in which the psychotic problem with the metaphoric generation of meaning might be observed, concerns the comprehension and actual use of metaphor in speech. Lacan’s theory does not imply that individuals with psychosis cannot comprehend or adequately use metaphors at all. Rather, at precise points, peculiarities in metaphor use and comprehension can be observed. Indeed, based on Lacanian theory we, on the one hand, expect that situations in which a person is confronted with the desire of the other (“what do you want”) and/or the issue of one’s own existential identity (“who am I”) will entail *deficient metaphor comprehension:* metaphoric meanings implied in other people’s speech might not be comprehended and a literal interpretation of figuratively intended speech might stand to the fore. On the other hand, as speech is actively used in situations related to desire and existence, we expect *deficient metaphor use*: metaphors will be poorly used in defining who one is and how one is positioned in relation to the other. Both phenomena bear witness to concretism in language use.

An example can illustrate this. A. is a young woman consulting the second author. She suffers from schizophrenia. One day her previous therapist told her to “see the world differently,” and to “look at her relational problems through the eyes of her boyfriend.” More than a year later she is still confused about these words. She feels “pressure on her eyes,” which she seems to experience physically, and she makes fun of her therapist. “I can only see the world through my own eyes… what should I look at?” A. is not affected by the empathy-oriented meaning implied in her therapist’s visual metaphors. They don’t help her make sense of her relationship. However, starting from the therapist’s interventions, she often lingers on the idea of her boyfriend’s eyes: “I sometimes think: ‘he is only eyes, as if he has no body anymore… Can you marry a pair of eyes?”

A second phenomenon in which the central problem with the metaphoric generation of meaning is often observed, is *autonymic speech*. In line with what classic psychiatrists like Kraepelin, Bleuler and de Clérambault already stressed, Lacan (1955–1956) indicates that in psychotic patient’s speech neologisms might be observed. In such cases “certain words take on a special emphasis, a density that sometimes manifests itself in the very form of the signifier, giving it this frankly neologistic character that is so striking in the creations of paranoia” (Lacan, 1955–1956, p. 32).

Several authors have examined the use of neologisms in schizophrenia and related disorders (McKenna, 2007). In psychiatry, a neologism is defined “as a completely new word or phrase whose derivation cannot be understood” (Andreasen, 1986). Such an idiosyncratic use of words is present in acutely psychotic schizophrenic patients, but tends to persist during partial remission and is unusual in non-schizophrenics (Harrow et al., 1972, 1973). Characteristically, in his discussion of neologisms Lacan argues that it is more specifically people’s *use* of language that should be taken into account. What matters in the neologistic character of certain words is not so much the question as to whether they are lexically new or not, but the fact that they are “autonymous” or self-referential (Lacan, 1959–2006, p. 450). Lacan characterizes them as “lead in the net” (Lacan, 1955–1956, p. 33), as immobile “erotized” elements in the network of the subject’s discourse (Lacan, 1955–1956, pp. 54–55). In other words, neologisms are a peculiar type of signifier, which present themselves as isolated elements that do not enter the referential process with other signifiers. Lacan concludes that they are the hallmark of delusions (Lacan, 1955–1956, p. 33–34). In contrast to a metaphor, autonyms are not the result of a creative move by the speaker, but words or sentences that were revealed (Lacan, 1955–1956, p. 85). Autonyms name an otherwise not-nameable reality, and cannot be brought into dialog with other elements of a person’s speech. Again, starting from Lacan’s overall psychosis theory, we expect that the active use of autonymous speech elements will especially come to the fore in situations in which a person is confronted with the enigma of the desire of the other (“what do you want?”) and/or the issue of one’s own existential identity (“who am I?”). The more intense such a confrontation is, the more prevailing autonymous speech will be.

For example, F. is in his 30s when his first psychotic episode occurs. After years of traveling around the world F. comes home to visit his mother, who has a life-threatening disease and no hope of recovery. One day, as he visits his mother at the intensive care department, he sees through the window of mother’s room his father and brother speaking with a physician. Soon thereafter his mother dies, and F. concludes that on that very day, the “conspiracy” began: he believes that his father and bother made a decision with the physician about his mother’s death. Since that day he is no longer safe, and became the object of a conspiracy himself. With the word “conspiracy” F. denotes an element of evil that threatens to annihilate him. “Conspiracy” names what radically overwhelms him, but does not signify his position as a subject vis-à-vis his relatives and the medical world. In terms of Lacan’s theory, the signifier “conspiracy” is an autonymous element in F.’s speech.

A third language-use related phenomenon we consider to be characteristic of psychosis is the dominance of associations at the level of the signifier over associations at the level of the signified in daily life contexts. Generally, Lacan assumes that at the level of the unconscious the signifier is predominant over the signified: linguistic units are mainly connected because of similarities at the level of the signifier. At the level of the conscious ego the reverse is true: language is mainly used to convey ideas. In neurosis, where the unconscious is repressed, the predominance of the signifier only comes to the fore in specific phenomena of mental life: symptoms and so-called productions of the unconscious, like dreams, jokes and parapraxes. In psychosis, by contrast, the unconscious is not repressed (Bazan, 2007, 2012). As a result, it can be expected that in daily life psychotic individuals will be more strongly inclined to focus on associations between signifiers. The following example illustrates this. G. is a young English boy living in Italy. He has suffered from paranoid schizophrenia since the death of his father, when he was 18 years old. He says that soon after the death of his father his mother once scolded him for staying out late all the time, shouting: “come here.” He believes that these English spoken words actually have a sexual meaning, and imply that his mother wanted him to ejaculate at home. Both in Italian than in English, the words “come here” are ambiguous and might also refer to ejaculation. In this case the common sense signified of “come here,” which situates his mother in a punishing role, could not be heard. What occupies his mind, and cannot be repressed, is the sexual meaning related to the Italian expression vieni qui or to the English ‘come/cum. Whereas in neurosis such sexual meanings would be repressed, they are out in the open in psychosis. Consequently, G. came to believe that his mother “wanted him to ejaculate at home.” In line with Lacan’s theory it might be that the loss of his father brutally confronted G. with the inability to symbolize the desire of his just-widowed mother. The catastrophic result of this phenomenon is that the patient came to interpret the desire of his mother as a sexual desire.

## Neuro-Scientific Perspectives on the Role of Symbolization in Psychosis

From a neurobiological point of view, psychotic disorders are interpreted in different ways. For example, they have been considered as the result of a chronic hyperdopaminergic activity (Cantrup et al., 2012), of a NMDA receptor hypofunction (Coyle, 2012) or of an abnormal brain connectivity (Yang et al., 2014). The hypofunction of the NMDA receptors has been linked to the impairment of the synaptic plasticity and of the mechanism of the long-term potentiation in schizophrenia (Steullet et al., 2006). Within this neuro-scientific context, Tim Crow is the researcher who best hypothesized a pivotal involvement of the language in the onset of psychotic disorders (Crow, 1997). According to his hypothesis, psychotic symptoms are intrinsic to the human capacity for language and they represent “language at the end of its tether” (Crow, 1997), as they provide a window on the transition between speech and thought (Crow, 1998). More precisely, he points to a problem relating to hemispheric differentiation: as a consequence of failed hemispheric differentiation, psychotic subjects suffer from a loss of the distinction between thought and speech, and in particular of the distinction between self-generated messages, and those that he receives as a hearer. Crow (2000) suggests that in schizophrenia the neural framework necessary to this distinction is altered. More recently, Magaud et al. (2010) have reported that individuals at high risk of psychosis have difficulty in retrieving words in response to categories but not to letters. Crow et al. (2012) suggested that such category-letter discrepancy is a lead to the pathophysiology of schizophrenia. In his commentary to the article of Magaud et al. (2010), he postulated that the phonological engrams are segregated to one (the left) hemisphere while the primary meanings are in the opposite hemisphere (more complex associations may be in either hemisphere). As a consequence of reduced hemispheric differentiation, the problems of psychosis arise in the connections between phonological (intra-) and semantic (inter-hemispheric) representations, primarily in the dominant hemisphere (Crow et al., 2012). This might explain why subjects at high risk of psychosis are more impaired in the semantic fluency rather than in the phonological capacity (Magaud et al., 2010; Crow et al., 2012).

The problem with language processing in psychosis may be evaluated through two different aspects: metaphoric speech and concretism (Voth and Bradshaw, 1978). In the field of linguistics it has been suggested that the symptoms of schizophrenia are evidence of neither a thought disorder nor a syntactic discursive disorder, but of a semiotic disorder (Harrod, 1986). Semiotics is the study of signs and symbols; it includes semantics, which concerns the meanings of words and the *relations* between the words themselves and what *they mean*. Confusion between metaphorical and literal use of language and inability to understand “what is being talked about,” i.e., what the referent of the conversation is, suggests that schizophrenia might be conceptualized as a semiotic disorder. Metaphoric and suggestive speech require symbolic interpretations that go beyond the literal meaning of words. Therefore, the understanding of metaphor requires the ability to symbolize, i.e., to use and understand figurative speech.

Concretism represents a common tool for investigating an individual’s ability to symbolize. It is commonly defined as the inability to understand the figurative meaning of proverbs and metaphors (Kircher et al., 2007). However, concretism could be conceptualized more precisely as having the opposite meaning, i.e., the practice of representing abstract concepts or qualities in concrete form. An illustrative example of concretism was already formulated above in the case of A., who became confused by the visual metaphors of her therapist. Another example is the following: M., a 37-year-old schizophrenic patient. In order to understand what his mother thought (“had in mind”) of him, he smashed her skull to look inside. In this case, an abstract concept (“to have in mind”) failed to be understood figuratively, and is represented in a very concrete form. Another possible explanation of this example may be provided by the “literalization” hypothesis, according to which delusions may represent a metaphoric idea about aspects of the world or the self and are expressed in a literal way (Elvevåg et al., 2011). This closely connects with Freud’s (1915) suggestion that in psychosis, figurative meanings are taken literally. In the same vein, Rhodes and Jakes (2004) demonstrated that deficient metaphoric speech might make a contribution to delusional content. In some individuals it is present before plain psychotic symptoms come to the fore, when extreme views of self, others and the world are created. For example, there may occur a disturbing thought in which a metaphoric expression describing the self is taken literally, such that it crystallizes new or previously existing negative emotions and thoughts (Rhodes and Jakes, 2004).

## Studies on Metaphoric Speech Processing in Psychosis

Impairments in metaphor-processing are commonly observed in schizophrenia (Mitchell and Crow, 2005, see Table 1 for an overview of the current literature on this topic). A recent study demonstrated that compared to control subjects, psychotic patients interpret metaphors more literally (Elvevåg et al., 2011). Interestingly, it has previously been demonstrated that schizophrenic patients show reduced priming for literally plausible idioms but intact priming for literally implausible idioms compared with controls (Titone et al., 2002). Consequently it could be hypothesized that psychotic patients have fewer difficulties in the case of literally implausible idioms, i.e., when no other interpretation of the idiom itself is possible than the literal one. In fact, individuals with high scores at the Schizotypal Personality Questionnaire (SPQ) and healthy controls do not differ in their ability to discriminate between appropriate and inappropriate statements, whether literal or metaphoric (Humphrey et al., 2010). Another study found that there is no difference between high-schizotypal adults and low-schizotypal adults in terms of identifying appropriate metaphors (Langdon and Coltheart, 2004). Recently, it has been shown that the interpretation of figurative language, such as metaphors, does not depend only on semantic and syntactical decoding but may also require non-linguistic abilities such as the appreciation of other mental states (e.g., knowledge, intention, and belief). Furthermore, it may depend on the salience of idiomatic meaning (familiarity, conventionality, and frequency of use; Iakimova et al., 2010). Interestingly, however, it does not depend on IQ (Mo et al., 2008). Although other aspects apart from the linguistic one may influence the comprehension of non-literal language, there is considerable evidence that impaired access to semantic codification plays an important role in schizophrenia. Interestingly, a recent study has observed a small positive relation between impairment in metaphor comprehension and negative symptomatology (Mossaheb et al., 2014). Finally, it is worthy of note that different studies have provided evidence of impaired proverb comprehension in schizophrenia (Brüne and Bodenstein, 2005; Thoma et al., 2009; Haas et al., 2014; Rapp et al., 2014).

## The Role of the Right Hemisphere in Metaphoric Speech

Despite the historical and acknowledged dominance of the left hemisphere in language, researchers have recently focused their attention on the right hemisphere. In particular, both clinical and research data (neuroimaging and EEG studies) suggest that the right hemisphere may play a crucial role in processing figurative language (Brownell et al., 1990; Rapp, 2009). In line with this, a recent meta-analysis (Yang, 2012) contends that the right hemisphere is responsible for processing coarse semantic information in language comprehension. In particular, the right hemisphere may play an important role in activating broad semantic fields and integrating concepts that may have distant semantic relations. The areas mostly involved are the right fronto-temporal regions, including inferior frontal gyrus, middle frontal gyrus, insula, superior temporal gyrus, and middle temporal gyrus (Yang, 2012).

However, right-hemisphere mechanisms may be necessary but not sufficient, for clarifying patients’ understanding of novel, low salience metaphoric expressions. The involvement of other brain areas than the right hemisphere in the comprehension of novel metaphors has been supported by both studies involving people with brain damage (Giora et al., 2000; Zaidel et al., 2002) and healthy adults in neuroimaging studies (Rapp et al., 2004; Lee and Dapretto, 2006; Stringaris et al., 2007). Recently, fMRI studies have shown that the process of conventionalization of novel metaphors causes a specific increase in activity within the bilateral inferior prefrontal cortex, left posterior middle temporal gyrus, and right postero-lateral occipital cortex (Schmidt et al., 2012). According to the graded salience hypothesis (GSH) textual comprehension is influenced by salience, with more salient words, phrases, and sentences being easier to process and requiring less right-hemisphere recruitment than less salient text (Laurent et al., 2006).

## Right-Hemisphere Dysfunction and Metaphoric Speech in Psychosis

Several studies have highlighted a dysfunction of the right hemisphere in schizophrenia (Cutting, 1992; Mitchell and Crow, 2005). In particular, right-hemisphere dysfunction is suggested to be responsible for several psychopathological impairments in schizophrenia, such as social cognition (de Achával et al., 2012), abnormal visuospatial perception (Ribolsi et al., 2013), volitional ocular motor control (Tu et al., 2010), prosodic comprehension (Mitchell and Crow, 2005), and auditory gating (Hirano et al., 2010). Furthermore, several studies have investigated the link between deficient language comprehension and right hemisphere dysfunction in the pathophysiology of schizophrenia (Kircher et al., 2002; Mitchell and Crow, 2005; Bleich-Cohen et al., 2009). In this regard, various studies in schizophrenic subjects document abnormalities regarding the brain areas involved in the comprehension of metaphoric speech. In particular, a severely reduced asymmetry of the right superior temporal sulcus (STS) at the base of Heschl’s gyrus has been observed (Chance et al., 2008), and functional and structural MRI studies have shown a dysfunction involving the inferior frontal gyrus, the middle frontal gyrus and the right temporoparietal junction (de Achával et al., 2012).

By contrast, few brain studies have directly examined metaphoric processing in schizophrenia. Iakimova et al. (2005) demonstrated that schizophrenic patients exhibit a negative event related potential (ERP) N400 amplitude for both literal and metaphoric sentences. The amplitude of the ERP N400 is proportional to the difficulty of interpretation of figurative language, and might represent an index of brain activation while integrating the meaning of a stimulus in an appropriate context (Debruille, 2007; Lai et al., 2009). These results may suggest that schizophrenic patients are less efficient in integrating the semantic context of all sentences (Iakimova et al., 2005). In a study by Kircher et al. (2007), during the comprehension of metaphoric vs. literal speech, schizophrenia patients exhibited less activation than control subjects in the right posterior temporal cortex. The authors hypothesize that this dysfunction might underlie the clinical symptom of concretism, reflected in the impaired understanding of non-literal, semantically complex language structures. More recently, schizophrenia patients proved to have reversed lateralization during novel metaphor processing. In particular, they overactivated the left inferior frontal gyrus, whereas healthy participants had stronger signal changes in the right superior/middle temporal gyrus. A possible explanation of increased BOLD response in some left hemisphere areas is that patients recruit additional cognitive resources (e.g., working memory) in order to increase their comprehension of both novel and conventional metaphors (Mashal et al., 2013). These results suggest that the inefficient processing of novel metaphors in schizophrenia involves compensatory recruitment of additional brain regions than the right ones (Mashal et al., 2013). Finally, a recent study has explored metaphor-processing in patients affected by Asperger Syndrome, finding lesser activation of the right hemisphere during the comprehension of novel metaphors in these patients than in healthy subjects (Gold and Faust, 2010; Gold et al., 2010). These data suggest the hypothesis that right hemisphere-dependent difficulties in figurative language may be a core feature of autism spectrum disorders as well.

## Conclusion

The possibility of explaining psychosis in terms of a severe lack of symbolization has been supported by several psychoanalytic authors. Jacques Lacan, in particular, suggests that in psychosis a specific signifier, which concerns both the law and naming, is absent and as a result the structure of the Symbolic is unstable. Lacan calls this signifier the “Name-of-the-Father,” and refers to its absence with the concept of “foreclosure.” Based on our review of Lacan’s theory we suggest that language use of psychotic patients might, at least, be distinctive in three particular ways:

First, psychotic patients are expected to be more impaired in the comprehension and actual use of metaphors in speech. In particular, we expect that in situations in which a person is confronted with the desire of the other (“what do you want?”) and/or the issue of one’s own existential identity (“who am I?”) will entail *deficient metaphor comprehension* and *deficient metaphor use*. Starting from Lacanian theory we do not expect a generalized deficiency in metaphor use, but peculiar metaphor use and concretism when desire and existence related issues are at stake.

Second, Lacanian theory suggests that psychotic speech is frequently *autonymic*, with a rich use of neologisms or auto-referential words. Neologisms are an unusual type of signifier, which present themselves as isolated elements that do not enter the referential process with other signifiers. Again we presume that the active use of autonymous speech elements will especially come to the fore in situations in which a person is confronted with issues related to desire or existence.

A third language-use related phenomenon we consider to be characteristic of psychosis is the dominance of associations at the level of the signifier over associations at the level of the signified in daily life contexts. In psychosis the unconscious is not repressed, as a result of which drive and jouissance laden topics (e.g., concerning sexuality and destruction) easily come to mind and might preoccupy psychotic individuals.

From a neurophysiological point of view, there is evidence of both structural and functional abnormalities regarding the brain areas involved in the comprehension of metaphoric speech. In particular, severely reduced asymmetry of the right superior temporal sulcus at the base of Heschl’s gyrus has been observed in schizophrenia (Chance et al., 2008) and furthermore functional and structural MRI studies have shown a dysfunction involving the inferior frontal gyrus, the middle frontal gyrus and the right temporoparietal junction, and the right posterior temporal cortex. Therefore, although highly speculative, it could be hypothesized that a dysfunction of the right hemisphere, a brain area strongly involved in the pathophysiology of schizophrenia, may be associated with the onset of this impairment. Interestingly, some studies have reported that both senses of an ambiguous word remain active in the right hemisphere, while the left hemisphere rapidly suppresses the inappropriate meaning, i.e., the meaning that should be avoided through common sense (Faust and Gernsbacher, 1996). A possible hypothesis is that the dysfunction of the right hemisphere impairs the ability to keep the alternative senses of figurative speech, with the result of a fixation of one delusional meaning.

Given the strong focus on comprehension of metaphoric speech in the field of neuro-scientific research we wonder how both fields of study might converge. Traditionally, psychoanalysis focuses on the singularity of a case-by-case approach, which enables contextualized comprehension of specific points in a patient’s functioning. Neuro-scientific research, in its turn, studies specific phenomena in samples of subjects, which focuses on generalization across individuals, assuming that their contexts will be largely similar. However, independent of their specific methodologies, both approaches aim to build general theories on psychosis. Lacan’s theory aims to guide psychoanalytic praxis with psychosis, neuro-scientific theory aims to explain the nature of psychosis. When asking the question of a possible convergence between both fields of research, these differences at the level of methodology and with respect to the aim and focus of theory should be taken into account.

Consequently, although highly stimulating, such a convergence can only be partial as a neuro-scientific translation of a number of Lacanian concepts, like foreclosure or Name-of-the-Father is not possible. Such concepts simply don’t fit the requirement of operationalization that characterizes empirical research. However, the three aspects of language use that we presume to be characteristic of psychosis could be tested within empirical research designs, and might well connect with current neuro-scientific research paradigms.

From our discussion of the current neuro-scientific literature, it emerges that several studies have investigated the processing of metaphoric speech in schizophrenia. Although far from conclusive, the available evidence suggests that schizophrenia patients are more impaired in the interpretation of metaphors than controls. Presuming that at this level peculiarities can be observed, we believe that the Lacanian focus on desire and existence-related issues might well be taken into account when studying metaphor use and comprehension. Such an investigation may not only validate Lacanian insight, but also refine neuro-scientific predictions.

Moreover, the current neuro-scientific literature has confirmed the increased use of neologisms as a fundamental marker of language-use in psychosis (Jampala et al., 1989). These are strongly and specifically associated with grammatical–phonological encoding performance (Barch and Berenbaum, 1996). For example, in the Scale for the Assessment of Thought, Language, and Communication (Andreasen and Grove, 1986), the amount of neologisms during the interview with the psychotic patient is one of the items that should be evaluated from the clinician to assess formal thought disorders. Yet, from a Lacanian point of view, the study of neologisms should not strictly focus on newly created words, but also pay attention to highly private interpretations of existing words, and again pay attention to the desire and existence-related experiential contexts in which these are produced.

Concerning the dominance of associations at the level of the signifier over associations at the level of the signified, in this manuscript we reported a clinical case (the case of G.) that may represent a common example of this phenomenon. Interestingly, there is some evidence that individuals at high risk of psychosis have difficulty in retrieving words in response to categories but not to letters and are more impaired in the semantic fluency rather than in the phonological capacity (Magaud et al., 2010). To this regard Crow et al. (2012) postulated that psychosis may arise from an abnormal connection between phonological (intra-) and semantic (inter-hemispheric) representations, as a consequence of reduced hemispheric differentiation. However, specific neuro-scientific investigations still lack on this aspect. In line with the case of G. we suggest taking into account the precise life context within which the dominance of associations at the level of the signifier come to the fore. Building on the Lacanian concept of the unconscious we presume that especially speech and thought about drive and jouissance related topics will bring this predominance to the fore.

Conversely we can ask the question as to how neuro-scientific research might inspire Lacanian psychoanalysis. Neuro-scientific studies suggest that right-hemisphere dysfunction is responsible for both problems in figurative speech comprehension and other psychopathological including social cognition, abnormal visuospatial perception, volitional ocular motor control, prosodic comprehension, and auditory gating. In psychoanalytic clinical work, attention might be paid to the question as to if and how dysfunctions in these domains cohere in patients’ experiences and functioning. Furthermore, the observation that metaphor comprehension is affected in Asperger syndrome, just like in schizophrenia, might stimulate contemporary Lacanian analysts to further work on the question of autism in relation to psychotic structure, focusing on specific language use patterns (Maleval, 2009).

However, the convergence between Lacanian psychoanalysis and neuroscience has its limits. For example, in his study of psychopathology Lacan strongly stresses psychical causality, meaning that processes of identification and ways in which the signifier is used in relation to the Other determine psychopathology (Lacan, 1947–2006, 1966–2006). Neuroscientists, by contrast, usually stress biological mechanisms at the basis of psychosis. At this point convergence is difficult. Lacan was interested in the structure of mental phenomena biological psychiatrists like de Clérambault described, but with respect to the crucial determining factors diverged from them in stressing subjective processes and structures instead of biological bases.

To conclude, there are, first of all, some “diagnostic” implications that might be drawn from this study. Current nosographic systems substantially neglect peculiarities of language use in psychosis. For example, the criteria of schizophrenia from DSM-5 do not take in account the processing figurative language, but only focus on “disorganized speech.” Therefore we consider the question of language use, and of metaphor use in particular, as the forgotten dimension in psychosis, suggesting that it should be more central to the diagnosis of psychosis. Second, it could be interesting to examine how peculiarities in language use change during the course of the psychotic episodes. For example, it would be extremely interesting to investigate if such impairment is already present before and during the first episode of psychosis, or whether it appears only later after the onset of the disorder. Finally, there are some implications for the treatment. It would be very interesting to evaluate the changeability of this phenomenon, and to examine if and how both pharmacological and psychological treatments have an effect on language use in psychosis.

### Conflict of Interest Statement

The authors declare that the research was conducted in the absence of any commercial or financial relationships that could be construed as a potential conflict of interest.

## Footnotes

1 Jakobson (1953–1971) connects the synchrony and diachrony to two types of aphasia, which might be the result of a stroke. The first type is the similarity disorder, in which patients experience problems selecting the right word for naming events or objects. This aphasia is characterized by a substitution deficiency and implies that metaphoric processes are affected. The second type is the contiguity disorder. This time the patient loses the capacity to combine linguistic elements, hence the idea that metonymy is deficient. In his year-long seminar on psychosis [Lacan (1955–1956)] discussed Jakobson’s work on aphasia, but never connected it to further speculation on mechanisms involved in aphasia. What he focused on was how metonymy and metaphor function in psychosis.

2 For example, in Shakespeare’s *Titus Andronicus* Saturninus, the new emperor of Rome, suggests that the heroic general Titus Andronicus is involved in the kidnapping of his own daughter, Lavinia. Titus is shocked by Saturninus’ accusation and cries out loud: “These words are razors to my wounded heart” (Act 1, Scene A, Verse 314). This sentence is a metaphor. By saying that Saturninus’ words are razors, the material qualities usually attributed to this object (e.g., “sharp,” “cuts,” and “wounds if not handled carefully”) are transferred to the concept “words.” Often, words are seen as ephemeral communications with no real impact on life. When Titus Andronicus says that they are razors, the opposite gets communicated: a fearless warrior like him does not get wounded by the swords and knives of his enemies, but by the false accusations of the man he fully trusted. As reader of the play we anticipate that following Saturninus’ accusation, anger and disappointment will be expressed. However, Titus Andronicus does not simply indicate that he is angry or shocked by Saturninus’ words (the barred S’ in the formula of metaphor), but says that words are wounding razors (S in the formula of metaphor), which implies that a physical quality is attributed to Saturninus’ words.

## References

[B1] Andreasen N. C. (1986). Scale for the assessment of thought, language, and communication (TLC). Schizophr. Bull. 12, 473–482. 10.1093/schbul/12.3.473 3764363

[B2] Andreasen N. C. Grove W. M. (1986). Thought, language, and communication in schizophrenia: diagnosis and prognosis. Schizophr. Bull. 12, 348–359. 10.1093/schbul/12.3.348 3764356

[B3] Barch, D. ,M., Berenbaum H. (1996). Language production and thought disorder in schizophrenia. J. Abnorm. Psychol. 105, 81–88. 8666714 10.1037//0021-843x.105.1.81

[B4] Bazan A. (2007). Des Fantômes dans la Voix. Une hypothèse neuropsychanalytique sur la structure de l’inconscient. Voix Psychanalytiques. Montreal: Liber.

[B5] Bazan A. (2012). From sensorimotor inhibition to freudian repression: insights from psychosis applied to neurosis. Front. Psychol. 3:452. 10.3389/fpsyg.2012.00452 23162501 PMC3498871

[B6] Benveniste E. (1966). Problèmes de Linguistique Générale. Paris: Gallimard.

[B7] Bleich-Cohen M. Hendler T. Kotler M. Strous R. D. (2009). Reduced language lateralization in first-episode schizophrenia: an fMRI index of functional asymmetry. Psychiatry Res. 171, 82–93. 10.1016/j.pscychresns.2008.03.002 19185468

[B8] Bleuler E. (1934). Handbook of Psychiatry. New York: Macmillan.

[B9] Brownell H. H. Simpson T. L. Bihrle A. M. Potter H. H. Gardner H. (1990). Appreciation of metaphoric alternative word meanings by left and right brain-damaged patients. Neuropsychologia 28, 375–383. 10.1016/0028-3932(90)90063-T 1692980

[B10] Brüne M. Bodenstein L. (2005). Proverb comprehension reconsidered–‘theory of mind’ and the pragmatic use of language in schizophrenia. Schizophr. Res. 75, 233–239. 10.1016/j.schres.2004.11.006 15885515

[B11] Cantrup R. Sathanantham K. Rushlow W. J. Rajakumar N. (2012). Chronic hyperdopaminergic activity of schizophrenia is associated with increasedΔFosB levels and cdk-5 signaling in the nucleus accumbens. Neuroscience 222, 124–135. 10.1016/j.neuroscience.2012.07.027 22820052

[B12] Chance S. A. Casanova M. F. Switala A. E. Crow T. J. (2008). Auditory cortex asymmetry, altered minicolumn spacing and absence of ageing effects in schizophrenia. Brain 131, 3178–3192. 10.1093/brain/awn211 18819990 PMC2724907

[B13] Coyle J. T. (2012). NMDA receptor and schizophrenia: a brief history. Schizophr. Bull. 38, 920–926. 10.1093/schbul/sbs076 22987850 PMC3446237

[B14] Crow T. J. (1997). Is schizophrenia the price that Homo sapiens pays for language? Schizophr Res. 28, 127–141. 10.1016/S0920-9964(97)00110-2 9468348

[B15] Crow T. J. (1998). Nuclear schizophrenic symptoms as a window on the relationship between thought and speech. Br. J. Psychiatry 173, 303–309. 10.1192/bjp.173.4.303 9926033

[B16] Crow T. J. (2000). Schizophrenia as the price that homo sapiens pays for language: a resolution of the central paradox in the origin of the species. Brain Res. Brain Res. Rev. 31, 118–129. 10.1016/S0165-0173(99)00029-6 10719140

[B17] Crow T. J. Collinson S. L. James A. C. (2012). Phonological versus semantic fluency: key to pathophysiology? Schizophr. Res. 135, 194–195. 10.1016/j.schres.2011.11.028 22189256

[B18] Cutting J. (1992). The role of right hemisphere dysfunction in psychiatric disorders. Br. J. Psychiatry 160, 583–588. 10.1192/bjp.160.5.583 1591569

[B19] de Achával D. Villarreal M. F. Costanzo E. Y. Douer J. Castro M. N. Mora M. C. (2012). Decreased activity in right-hemisphere structures involved in social cognition in siblings discordant for schizophrenia. Schizophr. Res. 134, 171–179. 10.1016/j.schres.2011.11.010 22137736

[B20] Debruille J. B. (2007). The N400 potential could index a semantic inhibition. Brain Res. Rev. 56, 472–477. 10.1016/j.brainresrev.2007.10.001 18001839

[B21] de Saussure F. (1916). Course in General Linguistics. (Open Court Classics). Paperback 1998. London: Duckworth (Original work published 1903).

[B22] Elvevåg B. Helsen K. De Hert M. Sweers K. Storms G. (2011). Metaphor interpretation and use: a window into semantics in schizophrenia. Schizophr. Res. 133, 205–211. 10.1016/j.schres.2011.07.009 21821395

[B23] Faust M. E. Gernsbacher M. A. (1996). Cerebral mechanisms for suppression of inappropriate information during sentence comprehension. Brain Lang. 53, 234–259. 10.1006/brln.1996.0046 8726535 PMC4426501

[B24] Fink B. (2007). Fundamentals of Psychoanalytic Technique—A Lacanian Approach for Practitioners. New York: Norton.

[B25] Freud S. (1915). The Unconscious. Complete Psychological Works of Sigmund Freud, Vol. 19. London: Hogarth Press, 159–204.

[B26] Giora R. Zaidel E. Soroker N. Batori G. Kasher A. (2000). Differential effects of right- and left-hemisphere damage on understanding sarcasm and metaphor. Metaphor Symb. 1, 63–83. 10.1080/10926488.2000.9678865

[B27] Gold R. Faust M. (2010). Right hemisphere dysfunction and metaphor comprehension in young adults with Asperger syndrome. J. Autism. Dev. Disord. 40, 800–811. 10.1007/s10803-009-0930-1 20054629

[B28] Gold R. Faust M. Goldstein A. (2010). Semantic integration during metaphor comprehension in Asperger syndrome. Brain Lang. 113, 124–134. 10.1016/j.bandl.2010.03.002 20359737

[B29] Haas M. H. Chance S. A. Cram D. F. Crow T. J. Luc A. Hage S. (2014). Evidence of pragmatic impairments in speech and proverb interpretation in schizophrenia. J. Psycholinguist. Res. [Epub ahead of print]. 10.1007/s10936-014-9298-2 24756919

[B30] Harrod J. B. (1986). Schizophrenia as a semiotic disorder. Schizophr. Bull. 12, 12–19. 10.1093/schbul/12.1.12 3961424

[B31] Harrow M. Harkavy K. Bromet E. Tucker G. J. (1973). A longitudinal study of schizophrenic thinking. Arch. Gen. Psychiatry 28, 179–182. 10.1001/archpsyc.1973.01750320019003 4684285

[B32] Harrow M. Tucker G. J. Adler D. (1972). Concrete and idiosyncratic thinking in acute schizophrenic patients. Arch. Gen. Psychiatry 26, 433–439. 10.1001/archpsyc.1972.01750230043008 5019880

[B33] Hirano Y. Hirano S. Maekawa T. Obayashi C. Oribe N. Monji A. (2010). Auditory gating deficit to human voices in schizophrenia: a MEG study. Schizophr. Res. 117, 61–67. 10.1016/j.schres.2009.09.003 19783406

[B34] Humphrey M. K. Bryson F. M. Grimshaw G. M. (2010). Metaphor processing in high and low schizotypal individuals. Psychiatry Res. 178, 290–294. 10.1016/j.psychres.2009.06.002 20493534

[B35] Iakimova G. Passerieux C. Denhière G. Laurent J. P. Vistoli D. Vilain J. (2010). The influence of idiomatic salience during the comprehension of ambiguous idioms by patients with schizophrenia. Psychiatry Res. 177, 46–54. 10.1016/j.psychres.2010.02.005 20207011

[B36] Iakimova G. Passerieux C. Laurent J. P. Hardy-Bayle M. C. (2005). ERPs of metaphoric, literal, and incongruous semantic processing in schizophrenia. Psychophysiology 242, 380–390. 10.1111/j.1469-8986.2005.00303.x 16008767

[B37] Jakobson R. (1953–1971). “Results of the conference of anthropologists and linguists,” in Selected Writings II: Word and Language, eds Jakobson R. (The Hague/Paris: Mouton), 554–567.

[B38] Jampala V. C. Taylor M. A. Abrams R. (1989). The diagnostic implications of formal thought disorder in mania and schizophrenia: a reassessment. Am. J. Psychiatry 146, 459–463. 10.1176/ajp.146.4.459 2929745

[B39] Jakobson R. (1971–1985). Selected Writings, Vol. 6., ed. Rudy S. (The Hague/Paris: Mouton).

[B40] Kircher T. T. Leube D. T. Erb M. Grodd W. Rapp A. M. (2007). Neural correlates of metaphor processing in schizophrenia. Neuroimage 34, 281–289. 10.1016/j.neuroimage.2006.08.044 17081771

[B41] Kircher T. T. Liddle P. F. Brammer M. J. Williams S. C. Murray R. M. McGuire P. K. (2002). Reversed lateralization of temporal activation during speech production in thought disordered patients with schizophrenia. Psychol. Med. 32, 439–449. 10.1017/S0033291702005287 11989989

[B42] Kraepelin E. (1913). Psychiatrie: ein Lehrbuch für Studierende und Arzte, 8th Edn. Leipzig: Barth Verlag. Selected chapters reprinted in translation as Dementia Praecox and Paraphrenia (1919) (ed. G. M. Robertson).

[B43] Lacan J. (1947–2006). “Presentation on psychical causality,” in Écrits: The First Complete Edition in English, ed. Miller J.-A. (New York and London: W.W. Norton & Company), 123–158.

[B44] Lacan J. (1953). “Le Symbolique, l‘Imaginaire et le Réel’,” in Des Noms-Du-Père, eds. Lacan J. Miller J.A. (Paris: Seuil), 9–63.

[B45] Lacan J. (1955–1956). “The other Psychose,” in The Seminar of Jacques Lacan, Book III: The Psychoses, 1955-1956, ed. Miller J.-A., trans. Russell Grigg (New York, NY: W.W. Norton), 1993.

[B46] Lacan J. (1956–1957). The Seminar of Jacques Lacan, 1956–1957, Book 4. La relation d’objet et les structures freudiennes (Seuil, 1994).

[B47] Lacan J. (1957–2006). “The instance of the letter in the unconscious or reason since Freud,” in Écrits, eds Lacan J. Miller J. A. (New York, NY: W. W. Norton), 412–442.

[B48] Lacan J. (1957–1958). The Seminar of Jacques Lacan 1957–1958, Book 5: Les formations de l’inconscient (Seuil, 1998).

[B49] Lacan J. (1958). “On a question prior to any possible treatment of psychosis,” in Écrits: The First Complete Edition in English, ed. Miller J.-A., trans. B. Fink (New York, NY: W.W. Norton & Company).

[B50] Lacan J. (1959–2006). “On a question prior to any possible treatment of psychosis,” in Écrits, eds Lacan J. Miller J. A. (New York, NY: W. W. Norton), 445–488.

[B51] Lacan J. (1960–2006). “The subversion of the subject and the dialectic of desire in the freudian unconscious,” in Écrits: The First Complete Edition in English, ed. Miller J.-A. (New York and London: W.W. Norton & Company). 671–702.

[B52] Lacan J. (1966–2006). “On my antecedents,” in Ecrits: The First Complete Edition in English, ed. Miller J.-A., trans. B. Fink (New York and London: W.W. Norton), 65–72.

[B53] Lacan J. (1975–1976). Le Séminaire 1975-1976, Livre XXIII, Le Sinthome. Paris: Seuil.

[B54] Lai V. T. Curran T. Menn L. (2009). Comprehending conventional and novel metaphors: an ERP study. Brain Res. 1284, 145–155. 10.1016/j.brainres.2009.05.088 19505446

[B55] Langdon R. Coltheart M. (2004). Recognition of metaphor and irony in young adults: the impact of schizotypal personality traits. Psychiatry Res. 125, 9–20. 10.1016/j.psychres.2003.10.005 14967548

[B56] Laurent J. P. Denhières G. Passerieux C. Iakimova G. Hardy-Baylé M. C. (2006). On understanding idiomatic language: the salience hypothesis assessed by ERPs. Brain Res. 1068, 151–160. 10.1016/j.brainres.2005.10.076 16388782

[B57] Lee S. S. Dapretto M. (2006). Metaphorical vs. literal word meanings: fMRI evidence against a selective role of the right hemisphere. Neuroimage 29, 536–544. 10.1016/j.neuroimage.2005.08.003 16165371

[B58] Magaud E. Kebir O. Gut A. Willard D. Chauchot F. Olie J. P. (2010). Altered semantic but not phonological verbal fluency in young help-seeking individuals with ultra high risk of psychosis. Schizophr. Res. 123, 53–58. 20605416 10.1016/j.schres.2010.05.005

[B59] Maleval J. C. (2009). L’autiste, Son Double, et ses Objets. Rennes: PUR.

[B60] Mashal N. Vishne T. Laor N. Titone D. (2013). Enhanced left frontal involvement during novel metaphor comprehension in schizophrenia: evidence from functional neuroimaging. Brain Lang. 124, 66–74. 10.1016/j.bandl.2012.11.012 23291493

[B61] McKenna P. J. (2007). Schizophrenia and Related Syndromes. Paperback.

[B62] Mitchell R. L. Crow T. J. (2005). Right hemisphere language functions and schizophrenia: the forgotten hemisphere? Brain 128, 963–978. 10.1093/brain/awh466 15743870

[B63] Mo S. Su Y. Chan R. C. Liu J. (2008). Comprehension of metaphor and irony in schizophrenia during remission: the role of theory of mind and IQ. Psychiatry Res. 157, 21–29. 10.1016/j.psychres.2006.04.002 17854910

[B64] Mossaheb N. Aschauer H. N. Stoettner S. Schmoeger M. Pils N. Raab M. (2014). Comprehension of metaphors in patients with schizophrenia-spectrum disorders. Compr. Psychiatry 55, 928–937. 24556517 10.1016/j.comppsych.2013.12.021

[B65] Naveau P. (2004). Les psychoses et le lien social: Le noeud défait. Paris: Anthropos.

[B66] Nobus D. (2000). Jacques Lacan and the Freudian Practice of Psychoanalysis. New York: Routledge.

[B67] Parnas J. (2011). A disappearing heritage: the clinical core of schizophrenia. Schizophr. Bull. 37, 1121–1130. 10.1093/schbul/sbr081 21771902 PMC3196960

[B68] Rapp A. (2009). “The role of the right hemisphere for language in schizophrenia,” in Language lateralization in psychosis, eds Sommer I. E. Kahn R. S. 147–156. 10.1017/CBO9780511576744.011

[B69] Rapp A. M. Langohr K. Mutschler D. E. Wild B. (2014). Irony and proverb comprehension in schizophrenia: do female patients “dislike” ironic remarks? Schizophr. Res. Treatment. 2014, 10. 10.1155/2014/841086 24991434 PMC4060160

[B70] Rapp A. M. Leube D. T. Erb M. Grodd W. Kircher T. T. (2004). Neural correlates of metaphor processing. Brain Res. Cogn. Brain Res. 20, 395–402. 10.1016/j.cogbrainres.2004.03.017 15268917

[B71] Remington G. Agid O. Foussias G. (2011). Schizophrenia as a disorder of too little dopamine: implications for symptoms and treatment. Expert Rev. Neurother. 11, 589–607. 10.1586/ern.10.191 21469931

[B72] Ribolsi M. Lisi G. Di Lorenzo G. Koch G. Oliveri M. Magni V. (2013). Perceptual pseudoneglect in schizophrenia: candidate endophenotype and the role of the right parietal cortex. Schizophr. Bull. 39, 601–607. 10.1093/schbul/sbs036 22419195 PMC3627750

[B73] Rhodes J. E. Jakes S. (2004). The contribution of metaphor and metonymy to delusions. Psychol. Psychother. 77, 1–17. 10.1348/147608304322874227 15025901

[B74] Schmidt G. L. Cardillo E. R. Kranjec A. Lehet M. Widick P. Chatterjee A. (2012). Not all analogies are created equal: associative and categorical analogy processing following brain damage. Neuropsychologia 50, 1372–1379. 10.1016/j.neuropsychologia.2012.02.022 22402184 PMC3384712

[B75] Schreber D. P. (1955). Memoirs of My Nervous Illness , trans. I. Macalpine and R. A. Hunter. London: Dawson. (Original work published 1903).

[B76] Steullet P. Neijt H. C. Cuénod M. Do K. Q. (2006). Synaptic plasticity impairment and hypofunction of NMDA receptors induced by glutathione deficit: relevance to schizophrenia. Neuroscience 137, 807–819. 10.1016/j.neuroscience.2005.10.014 16330153

[B77] Stringaris A. K. Medford N. C. Giampietro V. Brammer M. J. David A. S. (2007). Deriving meaning: Distinct neural mechanisms for metaphoric, literal, and non-meaningful sentences. Brain Lang. 100, 150–162. 10.1016/j.bandl.2005.08.001 16165201

[B78] Thoma P. Hennecke M. Mandok T. Wähner A. Brüne M. Juckel G. (2009). Proverb comprehension impairments in schizophrenia are related to executive dysfunction. Psychiatry Res. 170, 132–139. 10.1016/j.psychres.2009.01.026 19906437

[B79] Titone D. Holzman P. S. Levy D. L. (2002). Idiom processing in schizophrenia: literal implausibility saves the day for idiom priming. J. Abnorm. Psychol. 111, 313–320. 10.1037/0021-843X.111.2.313 12003452

[B80] Tu P. Buckner R. L. Zollei L. Dyckman K. A. Goff D. C. Manoach D. S. (2010). Reduced functional connectivity in a right-hemisphere network for volitional ocular motor control in schizophrenia. Brain 133, 625–637. 10.1093/brain/awp317 20159769 PMC2858012

[B81] Van Haute P. (2001). Against Adaptation: Lacan’s Subversion of the Subject.

[B82] Vanheule S. (2011). The Subject of Psychosis: A Lacanian Perspective. Hampshire: Palgrave Macmillan. doi: 10.1057/9780230355873

[B83] Verhaeghe P. (2004). On Being Normal and Other Disorders: A Manual for Clinical Psychodiagnostics. New York, NY: Other Press.

[B84] Ver Eecke W. (2006). Denial, Negation, and the Forces of the Negative. Freud, Hegel, Lacan, Spitz, and Sophocles. Albany: State University of New York Press.

[B85] Voth H. M. Bradshaw S. Jr. (1978). Metaphor as a diagnostic tool. J. Clin. Psychiatry 39, 670–672. 681306

[B86] Yang J. (2012). The role of the right hemisphere in metaphor comprehension: a meta-analysis of functional magnetic resonance imaging studies. Hum. Brain Mapp. Aug. [Epub ahead of print]. 22936560 10.1002/hbm.22160 PMC6868953

[B87] Yang G. J. Murray J. D. Repovs G. Cole M. W. Savic A Glasser M. F. (2014). Altered global brain signal in schizophrenia. Proc. Natl. Acad. Sci. U.S.A. 111, 7438–7443. 10.1073/pnas.1405289111 24799682 PMC4034208

[B88] Zaidel E. Kasher A. Soroker N. Batori G. (2002). Effects of right and left hemisphere damage on performance of the “Right Hemisphere Communication Battery”. Brain Lang. 80, 510–535. 10.1006/brln.2001.2612 11896655

